# Intracranial Major Artery Stenosis Associated With Acute Posterior Multifocal Placoid Pigment Epitheliopathy

**DOI:** 10.7759/cureus.88147

**Published:** 2025-07-17

**Authors:** Kosuke Suzuki, Kaori Sumi, Teruaki Masuda, Ryoko Oki, Atsunobu Takeda, Noriyuki Kimura

**Affiliations:** 1 Neurology, Faculty of Medicine, Oita University, Yufu, JPN; 2 Ophthalmology, Faculty of Medicine, Oita University, Yufu, JPN; 3 Ophthalmology, Nagatomi Neurosurgery Hospital, Yufu, JPN

**Keywords:** acute posterior multifocal placoid pigment epitheliopathy, antiplatelet agents, immunotherapy, intracranial major artery stenosis, juvenile cerebral infarction, prednisolone

## Abstract

Acute posterior multifocal placoid pigment epitheliopathy (APMPPE) is an immune-mediated chorioretinal disease that can cause cerebral infarctions in young individuals. We report the case of a 16-year-old girl who developed intracranial major artery stenosis. She presented with eye pain and microcerebral infarctions, and CSF examination showed lymphocytosis and elevated interleukin-6 (IL-6). Ophthalmoscopy revealed multiple white posterior pole lesions; hence, she was diagnosed with cerebral infarction due to APMPPE. Five weeks after the initiation of prednisolone (PSL) treatment, she developed asymptomatic intracranial arterial stenosis. The addition of intravenous methylprednisolone (IVMP) and antiplatelet drugs resulted in the complete resolution of arterial stenosis and neurological symptoms.

It is important to recognize APMPPE as a potential cause of juvenile cerebral infarction accompanied by ocular symptoms. In addition, careful monitoring for intracranial major artery stenosis is warranted, as vascular stenosis may progress despite immunotherapy.

## Introduction

Acute posterior multifocal placoid pigment epitheliopathy (APMPPE), first described by Gass in 1968, is an inflammatory chorioretinopathy characterized by acute visual symptoms and yellowish-white placoid lesions at the level of the retinal pigment epithelium, located primarily in the posterior pole, and occurs in young individuals aged 20-40 years [1,2]. Most cases of APMPPE present only with ocular symptoms and resolve within two-three months [2]. However, it has been reported that APMPPE is sometimes accompanied by central nervous system involvement, such as stroke [2-5]. Cerebral vasculitis has been proposed as a mechanism of cerebral infarction associated with APMPPE based on histological findings and CSF pleocytosis [3,6-10].

Here, we report the case of a 16-year-old girl who presented with cerebral infarction and major intracranial artery stenosis associated with APMPPE. She was treated with oral prednisolone (PSL) but developed asymptomatic intracranial major artery stenosis. The patient responded favorably to intravenous methylprednisolone (IVMP) and cilostazol. This report aims to provide insights into the management and treatment of stroke associated with major intracranial artery stenosis in patients with APMPPE.

## Case presentation

**Figure 1:**
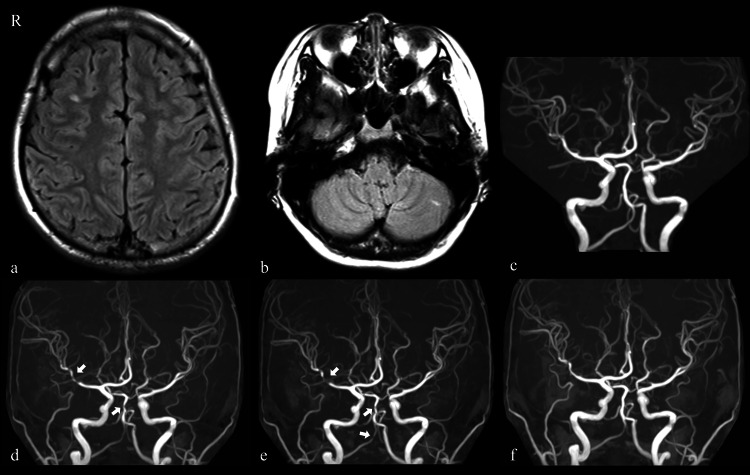
Imaging findings of the patient a-c: MRI of the head revealed multiple cerebral infarctions in the right frontal lobe and left cerebellar hemisphere, with no evidence of vascular stenosis. d: MRA performed on day 34 showing stenosis of the right MCA and BA (arrows). e: By day 47, stenosis of the MCA and BA had progressed, and new stenosis was observed in the right posterior cerebral and right vertebral arteries (arrows). f: The MRA findings normalized by day 52. BA: Basilar artery; MCA: Middle cerebral artery; MRA: Magnetic resonance angiography

A 16-year-old Japanese girl presented to a local clinic with transient dizziness, vomiting, headache, right eye pain, diplopia, and numbness of her lips and left extremities. MRI of the head revealed multiple acute small cerebral infarctions in the right frontal lobe and left cerebellar hemisphere, without evidence of cerebrovascular stenosis (Figure 1a, 1b, 1c), and she was referred to Oita University one week after onset.

**Figure 2:**
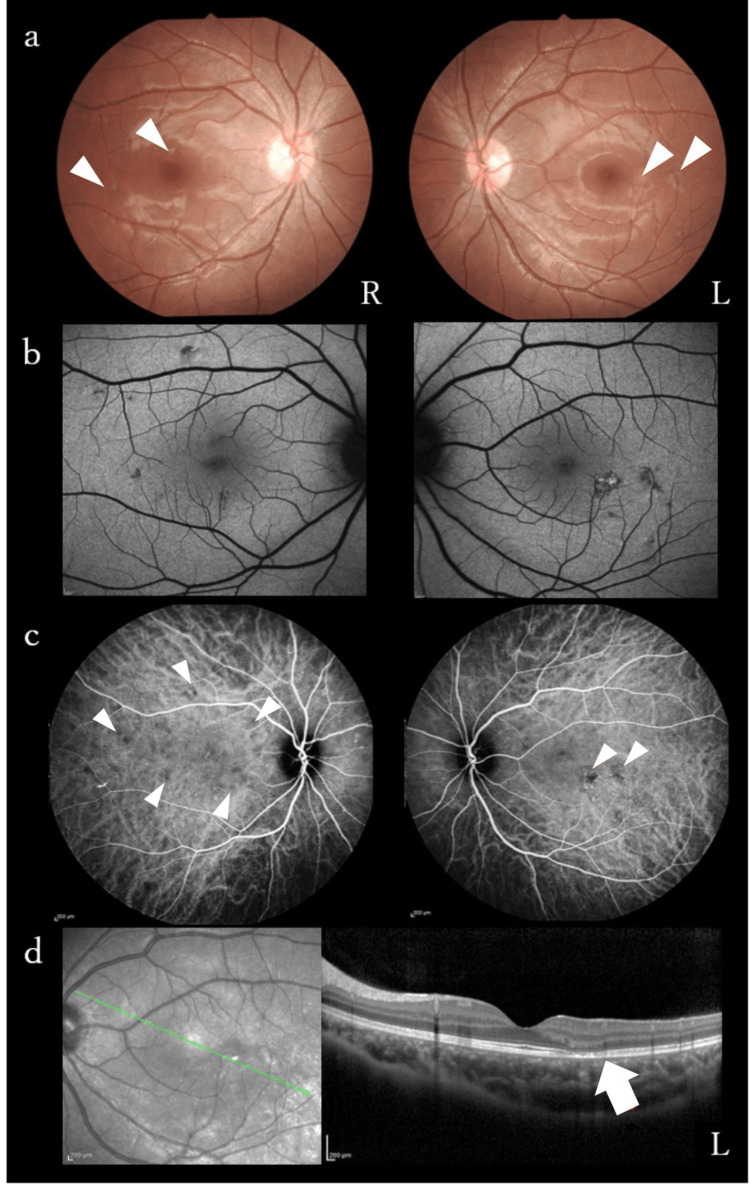
Multimodal ocular imaging findings of the patient a, b: Ophthalmoscopy and fundus autofluorescence revealed scattered scars of retinal pigment epithelium in the posterior pole (arrowheads). c: Indocyanine green angiography revealed punctate hypocyanescent lesions from the early to late phases, suggesting occlusion of the choriocapillaris (arrowheads). d: Optical coherence tomography of the left eye showed outer retinal architectural disruption (arrow) and thickened choroid with dilated vessels.

The patient had no risk factors for cerebral infarction or relevant family history. Physical examination revealed mild weakness and deep sensory disturbance of the left lower extremity, without headache or fever. Laboratory examination revealed a normal coagulation system with no elevation in C-reactive protein or white blood cell count (WBC). She was negative for antinuclear antibodies and weakly positive for immunoglobulin G (IgG) anticardiolipin antibodies at 13 U/mL (≤ 12U/mL), but the repeat test was negative. CSF analysis showed lymphocytic pleocytosis (26 cells/mm^3^; normal values < 5 cells/mm^3^) and an elevated protein level (46.6 mg/dl; normal, ≤ 40.0 mg/dl). The CSF interleukin-6 (IL-6) level was as high as 109 pg/mL, IgG index was 0.52, and oligoclonal bands were negative. Electrocardiography and transesophageal echocardiography results were normal, and contrast-enhanced CT of the trunk presented no evidence of a thrombus or malignancy. We performed an ophthalmologic evaluation three weeks after the initial symptoms because the patient had ocular symptoms at the onset of the stroke. Ophthalmoscopy, fundus autofluorescence, and optical coherence tomography revealed scattered scars extending from the outer retina to the retinal pigment epithelium of the posterior pole in both eyes (Figure 2a, 2b, 2d). Late phase indocyanine green angiography revealed multiple hypocyanescent lesions due to occlusion of the choriocapillaris (Figure 2c). APMPPE, multiple evanescent white dot syndrome (MEWDS), and punctate internal choroidopathy (PIC) were considered as differential diagnoses for hypofluorescent lesions confined to the posterior pole in young women. MEWDS was excluded due to the bilateral presentation, and PIC was deemed unlikely because of the absence of well-circumscribed, small, round exudative lesions. Based on these findings, she was diagnosed with APMPPE in the inactive phase. 

The patient was diagnosed with cerebral infarction associated with APMPPE since there were no specific blood tests or imaging findings for other systemic small vessel occlusive vasculitis and was started on PSL 60 mg/day and warfarin therapy. 

CSF findings improved over time after the start of treatment; however, on day 34, magnetic resonance angiography (MRA) revealed stenosis of the right middle cerebral artery (MCA) and basilar artery (BA) without accompanying clinical symptoms (Figure 1d). In the early phase following treatment with IVMP, stenosis of the MCA and BA progressed, and new stenotic lesions developed in the right posterior cerebral artery (PCA) and right vertebral artery (VA) (Figure 1e). However, with continued oral PSL and a switch in antithrombotic therapy from warfarin to cilostazol in anticipation of vasodilatory effects, the cerebrovascular stenosis resolved by day 52 (Figure 1f). Neurological symptoms resolved completely, and the patient was discharged on day 56. Cilostazol was discontinued on day 167, and oral PSL was gradually tapered over two years.

## Discussion

Here, we describe a case of APMPPE presenting with cerebral infarction and major intracranial artery stenosis. This case demonstrates that APMPPE should be considered in juvenile patients with both cerebral infarction and ocular symptoms. Furthermore, cerebrovascular disease can be exacerbated during immunotherapy, and a combination therapy with PSL and antiplatelet agents may be useful. These findings provide insights into the management and treatment of stroke patients with APMPPE.

This case was characterized by a vascular lesion affecting medium-sized vessels rather than small vessels. CNS vasculitis associated with APMPPE mainly involves the small arteries and less commonly affects medium-sized vessels, as in the present case [6,11]. Therefore, we reviewed the literature for case reports of patients with APMPPE who developed intracranial medium-sized artery stenosis. We identified 12 cases through searches of PubMed and Google Scholar up to March 2025 using the search terms "APMPPE," "cerebral vasculitis," "cerebral infarction," and "stroke" [4,6-9,11-16]. A summary of the 13 cases, including ours, is presented in Table 1.

**Table 1:** Clinical characteristics of APMPPE patients with intracranial major artery stenosis ACA: Anterior cerebral artery; APMPPE: Acute posterior multifocal placoid pigment epitheliopathy; AZA: Azathioprine; BA: Basilar artery; CPA: Cyclophosphamide; d: Day; F: Female; IVMP: Intravenous methylprednisolone; M: Male; MCA: Middle cerebral artery; NA: Not applicable; PCA: Posterior cerebral artery; PSL: Prednisolone; SCA: Superior cerebellar artery; VA: Vertebral artery; WBC: White blood cell count

Author (year)	Age/Sex	Timing of stroke after ocular symptoms	Stenotic vessels	CSF findings	Immunothrapy (maximum dose)	Exacerbation during immunotherapy/timing	Immunotherapy at the time of exacerbation	Outcome
Smith [12]	25/M	2 months	Right PCA	WBC 100 cells/mm^3^, protein 30 mg/dl	Oral PSL (60 mg/d)	(-)	NA	Decreased vision of both eyes
Wilson [7]	24/M	1 month	Multifocal	WBC 0 cells/mm^3^, protein 66 mg/dl	Oral PSL (40 mg/d)	(+)/1 day	Oral PSL (20 mg/d)	Died
De Vries [9]	23/M	3 days	Left MCA, right PCA	NA	(-)	(-)	NA	Died
Luneau [11]	43/M	1 month	Right ACA, MCA	WBC 253 cells/mm^3^, protein 57 mg/dl	IVMP (1,000 mg/d), oral PSL, CPA (150 mg/d), AZA (50 mg/d)	(+)/6 months	Oral PSL (10 mg/d), switching from CPA to AZA	Mild decrease in left ﬁnger movements
Volbers [8]	22/M	1 month	Left MCA	WBC 16 cells/mm^3^	Oral PSL, AZA	(-)	NA	Full recovery
Matamala [13]	15/M	1 year	Left PCA	WBC 13 cells/mm^3^	Oral PSL, AZA	(-)	NA	NA
Case [6]	23/M	5 months	Bilateral ACA	Normal	IVMP, oral PSL (80 mg/d)	(-)	NA	Left leg weakness
Algahtani [4]	26/F	3 months	Bilateral ACA, MCA	WBC 10 cells/mm^3^, normal protein	IVMP, oral PSL (80 mg/d)	(-)	NA	Full recovery
Tsuboyama [14]	55/M	3 weeks	Left ACA, right SCA, PCA	WBC 21 cells/mm^3^, protein 40 mg/dl	IVMP, oral PSL, CPA	(+)/5 months	Oral PSL (details are unclear)	Improvement
Tsuboyama [14]	64/M	1 month	Bilateral MCA, PCA and SCA, BA, right VA	NA	IVMP, oral PSL (80 mg/d), CPA	(+)/3 weeks	Oral PSL (60 mg/d)	Died
Maamari [15]	21/M	1 month	Multifocal	NA	Oral PSL (80 mg/d)	(+)/18 days	Oral PSL (80 mg/d)	Died
Oki [16]	29/M	1 month	Right PCA	WBC 16 cells/mm^3^, protein 53.7 mg/dl	Oral PSL (50 mg/d)	(-)	NA	Full recovery
Present case	16/F	Simultaneous	Right MCA, BA, right PCA, VA	WBC 26 cells/mm^3^, protein 46.6 mg/dl	IVMP, oral PSL (60 mg/d)	(+)/5 weeks	Oral PSL (60 mg/d)	Full recovery

As shown in Table 1, most patients with APMPPE and major cerebral artery stenosis were male (84.6%), with a median age of 29.7 years (range: 15-64 years). While ophthalmic APMPPE affects males and females equally, neurological complications have been reported to be more common in men, and our study showed a similar trend. All patients, except the present case, developed cerebral infarction after the onset of ocular symptoms. The median interval to stroke was nine weeks (range: 0-12 months); three patients experienced stroke more than three months after diagnosis, with the longest interval being 12 months. Immunotherapy with PSL was administered to 12 patients, five of whom also received immunosuppressants. In the present case, a new intracranial major artery stenosis developed despite immunotherapy. Notably, six patients (46.2%), including this patient, experienced worsening of cerebrovascular disease, including new infarcts or progressive arterial stenosis, following the initiation of immunotherapy. Of these cases, three deteriorated within one month, while the remaining three, including the present case, showed worsening beyond one month after treatment, up to six months [7,11,14,15]. All three cases of early exacerbations were fatal, whereas neurological outcomes were good in those who had exacerbations beyond one month. Four patients (30.8%) died of cerebrovascular disease and its complications. One patient did not receive immunotherapy, and one underwent rapid steroid tapering or discontinuation [7,9].

These findings suggest that the appearance of major intracranial arterial stenosis should be monitored for approximately one month after APMPPE diagnosis. CNS vasculitis associated with APMPPE usually occurs after the diagnosis of APMPPE, but as in our case, it can occur simultaneously with ocular symptoms; therefore, APMPPE should be considered when patients present with cerebral infarction and ocular symptoms. Furthermore, if arterial stenosis develops, the dose of immunotherapy should be maintained or adjusted carefully during the first month. In addition, vascular stenosis may progress even during immunotherapy; therefore, careful monitoring with head imaging, such as MRI, is recommended for approximately six months. In our case, the vascular stenosis progressed asymptomatically; therefore, regular monitoring with MRI was useful.

Antithrombotics were administered in combination with immunotherapy. For CNS vasculitis, European guidelines suggest adjunctive aspirin therapy for patients with medium-to-large vessel involvement, and one study has shown an association between aspirin and long-term remission [17,18]. The benefit of antithrombotic therapy in the cerebrovascular disease of APMPPE has not been proven; however, in this case and Algahtani's report, using antiplatelet agents, the patient recovered completely [4]. Adjunctive antiplatelet therapy might be beneficial in patients with APMPPE involving medium-sized arteries. Further clinical data are needed, since the optimal type of antiplatelet agent remains unclear.

A limitation of this study is the small number of cases. While it is important to note that findings from a few cases are not generalizable, the aim of this report is to promote caution in the management and treatment of APMPPE with intracranial major artery stenosis. In addition, the data included in this review are heterogeneous in terms of publication period, reporting detail, and diagnostic criteria, which may limit direct comparisons across cases. Furthermore, the definition of clinical outcomes such as “improvement” may differ across reports, introducing an additional layer of uncertainty to the outcome analysis. It remains unclear which cases of APMPPE develop medium-sized vasculitis rather than small-vessel involvement and which cases worsen during immunotherapy. To the best of our knowledge, no differences have been observed in the timing of vasculitis onset or CSF findings. Additional case reports are needed to improve our understanding of this condition.

## Conclusions

In conclusion, APMPPE is a cause of juvenile stroke with ocular symptoms and may have a fatal prognosis when associated with intracranial arterial stenosis. Cerebrovascular disease may be exacerbated during immunotherapy, requiring careful follow-up, such as by head MRI. The combination of immunotherapy and antiplatelet agents may improve clinical outcomes.
